# Chemopreventive glucosinolate accumulation in various broccoli and collard tissues: Microfluidic-based targeted transcriptomics for by-product valorization

**DOI:** 10.1371/journal.pone.0185112

**Published:** 2017-09-25

**Authors:** Young-Sang Lee, Kang-Mo Ku, Talon M. Becker, John A. Juvik

**Affiliations:** 1 Department of Medical Biotechnology, Soonchunhyang University, Asan, South Korea; 2 Division of Plant and Soil Sciences, West Virginia University, Morgantown, West Virginia, United States of America; 3 Department of Crop Sciences, University of Illinois at Urbana–Champaign, Urbana, Illinois, United States of America; Chungnam National University, REPUBLIC OF KOREA

## Abstract

Floret, leaf, and root tissues were harvested from broccoli and collard cultivars and extracted to determine their glucosinolate and hydrolysis product profiles using high performance liquid chromatography and gas chromotography. Quinone reductase inducing bioactivity, an estimate of anti-cancer chemopreventive potential, of the extracts was measured using a hepa1c1c7 murine cell line. Extracts from root tissues were significantly different from other tissues and contained high levels of gluconasturtiin and glucoerucin. Targeted gene expression analysis on glucosinolate biosynthesis revealed that broccoli root tissue has elevated gene expression of *AOP2* and low expression of *FMOGS-OX* homologs, essentially the opposite of what was observed in broccoli florets, which accumulated high levels of glucoraphanin. Broccoli floret tissue has significantly higher nitrile formation (%) and epithionitrile specifier protein gene expression than other tissues. This study provides basic information of the glucosinolate metabolome and transcriptome for various tissues of *Brassica oleracea* that maybe utilized as potential byproducts for the nutraceutical market.

## Introduction

*Brassica* crops have been domesticated by humans into morphotypes for which different tissues are used for consumption [1]. Therefore, only a portion of *Brassica* crop biomass is harvested and utilized for food and feed. For example, broccoli florets represent less than 10% of total aerial biomass of the plant [2]. Can residual biomass after harvest of *Brassica* vegetables be utilized to yield value-added co-products?

Broccoli and cauliflower production values in United States of America (USA) during 2015 were $1.035 billion and $371 million, respectively [3]. Broccoli production in 2015 was 17.6 million tons [3]. Assuming only 10% of broccoli is used for food, then about 158 million tons of by-product of broccoli was produced at 2015 in USA. Like broccoli, cauliflower and other *Brassica* crops also have considerable amounts of unused by-products after harvest. Despite the potential economic value of these by-products, there is little information available on the nutritional value and other potentially beneficial bioactivities of unharvested tissues of broccoli (leaves and roots) and collards (roots) due to limited interest in these non-edible tissues. However, these tissues can potentially be utilized as anti-bacterial, anti-nematodal, allelopathic (weed management) agents, or feedstocks for extraction of pharmaceutical products [4–6].

Glucosinolates are sulfur and nitrogen-containing secondary metabolites found primarily in the *Brassicaceae*. Glucosinolate biosynthesis occurs from amino acid precursors through chain elongation, core structure formation, and side-chain modifications [7]. Among these glucosinolates, biosynthesis of side-chain groups and subsequent modification is attributed to differences in bioactivity among the glucosinolates, and more specifically, their hydrolysis products. Sulforaphane (SF) is one of the most potent chemopreventive agents in broccoli, hydrolyzed from its precursor, glucoraphanin, by myrosinase [8]. The biosynthesis of glucoraphanin is derived from methionine and goes through multiple steps to generate the methylthioalkyl glucosinolates. A subclade of flavin-monooxygenases (*FMOGS-OX*_*1-5*_) catalyze the conversion of methylthioalkyl glucosinolates (e.g. glucoerucin) into methylsulfinylalkyl glucosinolates (e.g. glucoraphanin) [9]. An α-ketoglutarate-dependent dioxygenase called *AOP2* has been demonstrated to control the conversion of methylsulfinylalkyl glucosinolates (e.g. glucoraphanin) to alkenyl glucosinolates (e.g. gluconapin) [10]. Thus, in order to accumulate glucoraphanin, the precursor to SF, the gene expression level of *FMOGS-OX*_*1-5*_ and *AOP2* should theoretically be high and low, respectively.

Glucosinolates themselves are not bioactive compounds. It is their hydrolysis products, formed by the removal of the glucose moiety by the endogenous enzyme, myrosinase, that have been reported to possess many bioactivities, including cancer-prevention, biofumigation, and anti-microbial activities [11, 12]. The chemical structure of these hydrolysis products depends on the structure of the glucosinolate side-chain and reaction conditions such as pH, concentration of Fe^2+^, and presence/absence of specifier proteins, such as epithiospecifier protein (ESP) [13]. There are a number of potential specifier proteins, including the *epithiospecifier modifier 1* (*ESM1*) gene in *Arabidopsis*, which encodes a protein shown to inhibit function of ESP, leading to increased isothiocyanate production from glucosinolate hydrolysis [14]. In the absence of specifier proteins, the addition of Fe^2+^ ions also promotes nitrile formation [15]. Nitriles are weak chemopreventive compounds compared to the isothiocyanates like sulforaphane (SF) and phenethyl isothiocyanate (PEITC).

In the past few decades, research on glucosinolates in crop plants has increased following the discovery of their putative role as cancer-prevention agents, primarily due to their induction of phase II detoxification enzymes, among other beneficial bioactivities [16]. Quinone reductase (QR), a phase II enzyme, is present in mammalian tissues and has been used as a biomarker of anticarcinogenic activity from *Brassica* crops [17]. QR acts as a catalyst in the conversion of reactive and toxic quinones into stable and non-toxic hydroquinones, reducing oxidative cycling [18]. The health benefits of *Brassica* vegetables are strongly associated with hydrolysis products created from glucosinolates by endogenous myrosinase. Isothiocyanate hydrolysis products in particular, including SF, PEITC, erucin, and allyl isothiocyanate (AITC), have been reported as quinone reductase enzyme inducers [19, 20].

In this study, we hypothesize that the differences in glucosinolate composition between *B*. *oleracea* subspecies, as well as between the various tissues in a given subspecies, would correlate with changes in expression of genes associated with side-chain modifications. If we understand how this critical step of glucosinolate biosynthesis differs among various glucosinolate-containing plant species/subspecies and plant tissues, we may be able to manipulate glucosinolate composition for the benefit of the grower (reduced pest damage) or for human/animal health.

The objectives of this study were to determine the composition of glucosinolates, their hydrolysis products, and health promoting bioactivity among different tissues from various broccoli and collards cultivars. In addition, comparison of the gene expression and glucosinolate profiles was performed in an effort to provide important information about the genetic regulatory mechanism that may be used to manipulate glucosinolate concentration for the improvement of human health promoting bioactivity or other agronomic purposes. In order to address these objectives, we used microfluidic high-throughput reverse-transcription quantitative PCR (RT-qPCR) analysis using the Fluidigm system.

## Materials and methods

### Broccoli and collard cultivation

For this experiment, commercially available broccoli cultivars in the USA, ‘Arcadia’ and ‘Sultan’, were chosen (Sakata Seed America, Salinas, CA). Two broccoli accessions originating from Italy were obtained using the USDA Germplasm Resources Information Network (GRIN) database: ‘Broccoli Neri (PI662531)’, and ‘Broccoli Grande Precoce (PI662712). We also included a doubled haploid line called ‘VI-158’, originally procured from Dr. Mark Farnham at the USDA-ARS U.S. Vegetable Laboratory in Charleston, South Carolina. Five collard accessions were obtained from the USDA GRIN: PI143351 (originating from Iran), PI171531 (from Turkey), PI181720 (from Syria), PI204563 (Commonly cultivated in the USA), and PI662840 (from Portugal). In the transcriptomic analysis results, broccoli and collard varieties were coded in an effort to simplify the figure: B1 for ‘Arcadia’; B2 for ‘Broccoli Neri (PI662531)’; B5 for ‘VI-158’; C2 for ‘PI171531’; C3 for ‘PI181720’; C5 for ‘PI662840’.

Seeds for all *Brassica* crops were germinated in flats in the greenhouse facility at the University of Illinois at Urbana-Champaign filled with Sunshine^®^ LC1 professional soil mix (Sun Gro Horticulture, Vancouver, British Columbia, Canada) and were allowed to grow in the greenhouse for three weeks under a 25°C/18°C and 14 h/10 h day/night temperature/photoperiod regime with supplemental lighting. After three weeks, the flats were moved to raised beds outside to allow for acclimation to the outdoor environment before being transplanted into the field at the University of Illinois Vegetable Research Farm (40° 04′38.89″ N, 88° 14′ 26.18″ W). Transplanting took place between July 6 and July 20 in 2012. Varieties were grown in a randomized complete block design with three replicates. All plants were supplied with supplemental water via aerial irrigation as needed in the first 30 days after transplanting. Mechanical and hand weeding was done as needed. Various broccoli and collards tissues harvested as bulked samples from five whole plants were flash-frozen in liquid nitrogen, and transported on dry ice from the field to -20°C storage until those samples could be lyophilized. For sampling of broccoli florets, one quarter of the florets from each head were harvested from five plants for each biological replication. For sampling of the broccoli and collard leaves, three different leaf positions (one each from upper, middle, and bottom) were harvested from five plants for each biological replication. For sampling of broccoli and collard roots, whole taproots were harvested from five plants for each biological replication. After freeze-drying, samples were ground to a fine powder with coffee grinders and stored at -20°C until analyses were conducted.

### Analysis of glucosinolates

Glucosinolate contents were quantified according to Ku et al. [20] with slight modifications. Freeze-dried floret, leaf, and root powder samples (200 mg) were placed in a 15 mL centrifuge tube and mixed with 2 mL of 70% methanol. After heating at 95°C for 10 min in a heating block followed by cooling on ice, 0.5 mL of internal standard were added. For the internal standard, 1 mM sinigrin was used in the case of broccoli samples, while 1 mM glucosinalbin was used for collards samples. The absence of those internal standards in each corresponding crop was confirmed by preliminary experiments. After adding internal standards to the tubes, the tubes were vortexed, followed with centrifugation at 8,000 × g for 5 min at 4°C and the supernatants were collected. The pellets were re-extracted with 2 mL 70% methanol at 95°C for 10 min, and the supernatant was mixed with the previously collected supernatant. One mL of pooled extract was transferred into a 2 mL microcentrifuge tube (Fisher Scientific, Waltham, MA), and proteins were precipitated by adding 0.15 mL of a 1:1 mixture of 1 M lead acetate and 1 M barium acetate. After centrifuging at 12,000 × g for 1 min, all supernatants were loaded onto a column containing 1 M NaOH and 1 M pyridine acetate-charged DEAE Sephadex A-25 resin (GE Healthcare, Piscataway, NJ). Desulfation was conducted by adding arylsulfatase (*Helix pomatia* Type-1, Sigma-Aldrich, St. Louis, MO) for 18 h at room temperature, and the desulfo-glucosinolates were eluted with 3 mL ddH_2_O and filtered by 0.45 μm nylon syringe filter. The filtered sample (100 μL) was injected into an Agilent 1100 HPLC system (Agilent, Santa Clara, CA). Glucosinolates were separated by Kromasil RP-C18 column (250 mm × 4.6 mm) with mobile phase A (1% v/v acetonitrile containing 1mM ammonium acetate solution) and B (100% acetonitrile) under following gradient conditions: 0 min 0% B, 7 min 4% B, 20 min 20% B, 35 min 25% B, 36 min 80% B, 40 min 80% B, 41 min 0% B, detected at 229 nm, and a flow rates of 1 mL/min. The UV response factors for various glucosinolates have been published previously [21]. Typical HPLC chromatograms are available in S1 and S2 Figs.

### Analysis of glucosinolate hydrolysis products

The glucosinolate hydrolysis product content in floret, leaf, and root tissue of broccoli and collard were quantified according to Ku et al. [22] after slight modification. Freeze dried and powdered sample (50 mg) was placed in a 2 mL microcentrifuge tube and 1 mL of ddH_2_O was added. After 24 h of hydrolysis at room temperature in darkness followed by adding 150 μL of phenyl isothiocyanate (1 mg/mL, to check consistency of GC analysis), vortexing, and centrifugation at 12,000 × g for 5 min, 500 μL of the aqueous layer was taken and mixed with 500 μL of dichloromethane in a Teflon centrifuge tube. After vigorous vortexing for 30 s and centrifugation at 12,000 × g for 3 min, the dichloromethane layer containing glucosinolate hydrolysis products was transferred into a vial and analyzed by gas chromatography (Agilent 6890N, Agilent Technologies, Santa Clara, CA) equipped with flame ionization detector and HP-5 capillary column (30 m × 0.32 mm × 0.25 μm thickness). The injector and detector temperatures were 200°C and 280°C, respectively, and nitrogen was used for the carrier gas at a flow rate 6 mL/min in splitless mode. The oven temperature was initiated at 40°C, held for 5 min, increased up to 260°C at a rate of 10°C/min, and maintained for 10 min. Identification and quantification of each glucosinolate hydrolysis product was conducted by comparing the retention time and peak area to corresponding authentic standards. Agilent MSD (HP-5973N, Agilent) was used to get mass spectra with the same GC condition as above to confirm the peak identification with authentic standards (S1 Table) except for sulforaphane nitrile. In the case of 1-cyano-2,3-epithiopropane (CETP) and sulforaphane nitrile (SFN), relative response factors of 0.894 and 0.762 were calculated [23] by using AITC and SF as a reference (1.0), respectively.

### Quinone reductase (QR) inducing activity

Quinone reductase activity was measured according to Ku et al. [20] after slight modification, especially in preparing hydrolyzed plant extracts due to the presence of volatile and water-insoluble hydrolysis products in different tissues and crops. Freeze-dried sample powder (50 mg) was hydrolyzed in a 4 mL amber vial with polytetrafluoroethylene (PTFE) liner by adding 3.0 mL of ddH_2_O for 24 h at room temperature in darkness. In order to capture and carry volatile and water-insoluble hydrolysis products, 1 mL dimethysulfoxide (DMSO) was added and mixed by vortexing for 30 s. An aliquot (1.5 mL) was transferred into a microcentrifuge tube, and after centrifugation at 12,000 × g for 5 min, the supernatant plant extract was used for the QR assay. For the QR assay, hepa1c1c7 murine hepatoma cells (ATCC, Manassas, VA, USA) were cultivated in alpha-minimum essential medium (α-MEM) containing 10% fetal bovine serum, 100 U/mL penicillin and 100 μg/mL streptomycin in a CO_2_ incubator (37°C, 95% ambient air and 5% CO_2_). The cells were divided every other day. Prior to plant extract treatments, cells with 80–90% confluence were plated into 96-well plates (Costar 3595, Corning Inc, Corning, NY, USA) at a population density of 1 × 10^4^ cells per well, and incubated for 24 h. After removing α-MEM media, new media containing sample extracts [final concentration: 12.5 μg of freeze-dried sample in 200 μL of media] were added and incubated for a further 24 h. Growth media alone was used as a negative control. For the QR assay, treated cells were rinsed with phosphate buffer at pH 7.4, and then lysed with 50 μL 0.8% digitonin in 2 mM EDTA, incubated at 37°C for 10 min with agitation at 100 rpm. A 200 μL aliquot of reaction mixture [10 μM BSA, 82 μM Tween-20, 927 μM glucose-6-phosphate, 1.85 μM NADP, 57 nM FAD, 2 units of glucose-6-phosphate dehydrogenase, 725 nM 3-(4,5-dimethylthiazo-2-yl)-2,5-diphenyltetrazolium bromide (MTT), and 50 μM menadione (dissolved in acetonitrile) in 25 mM Tris buffer] was added to the lysed cells. Readings were made 5 times at every 50 s with a microplate reader (μQuant, Bio-Tek Instruments, Winooski, VT, USA) at 610 nm. Immediately after the final readings, 50 μL of 0.3 mM dicumarol in 25 mM Tris buffer was added, and the plate was read again (five time points, 50 s apart) to subtract basal MTT reduction non-specific to QR. Total protein contents were also measured according to the protocols provided by the BioRad assay (Bio-Rad, Hercules, CA, USA). Results for QR specific activity (nmol MTT reduced mg^/^min) were expressed as a ratio of treated to negative control cells.

### RNA extraction and quantitative real time-PCR

For transcriptomic studies, three varieties of broccoli (‘Neri’, ‘Arcadia’, and ‘VI-158’) and three accessions of collard (‘PI171531’, ‘PI181720’, and ‘PI662840’) which showed distinctive glucosinolate profiles compared to the others were selected. Total RNA was extracted from selected samples with the use of an RNeasy Mini Kit (QIAGEN) according to the manufacturer’s instructions. RNA quality was verified using an Agilent 2100 Bioanalyzer (Agilent Technologies) and was quantified using a NanoDrop 3300 spectrophotometer (Thermo Scientific, Waltham, MA). First-strand cDNA synthesis was performed with one μg of the total RNA using Superscript^™^ III First-Strand Synthesis SuperMix for qRT-PCR (Invitrogen, Carlsbad, CA, USA). Following synthesis, cDNA was diluted to 1/10 original concentration for use in all further analyses. Primers were designed using Primer Express software (Applied Biosystems, Foster City, CA, USA) from cDNA sequences annotated by previous publication [24]. These sequence data saved at http://www.OCRI-genomics.org and a database, Bolbase, were produced by the *Brassica oleracea* Genome Sequencing Consortium (BoGSC). The one exception to this were primers for *AOP2*, which were created from cDNA sequence obtained from NCBI (AY044425.1) due to unavailability of *AOP2* cDNA sequence on Bolbase. Some additional cDNA sequences from Bolbase not identified/annotated as glucosinolate-related genes in Liu et al. [24] were used for primer creation based on their BLAST bit-score for similarity to annotated *A*. *thaliana* sequences. Primer amplification efficiency and specificity was tested with the use of Power SYBR^®^ Green RT-PCR Master Mix (QIAGEN) and an ABI 7900HT Fast Real-Time PCR System (Applied Biosystems) according to the manufacturer’s instructions. Primer amplification efficiency was tested using a 5-fold serial dilution of bulk cDNA from all samples with the highest concentration being already 1/10 diluted from the original cDNA synthesis. Primer specificity was verified through dissociation curve analysis. The final list of primers, the gene model classifier from Bolbase, and the corresponding gene symbol used in this manuscript can be found in S2 Table. Following primer validation, cDNA samples and primers were submitted to the Roy J. Carver Biotechnology Center (University of Illinois at Urbana-Champaign, Urbana, IL, USA) for final transcript expression profiling using a Fluidigm Dynamic Array and Biomark HD high throughput amplification system (Fluidigm, South San Francisco, CA, USA) following 12 cycles of pre-amplification. Relative transcript quantification was performed using standard curves produced from bulk cDNA 5-fold serial dilutions. Data were normalized to each of the three endogenous control genes separately using the free Fluidigm Real-Time PCR Analysis software. Averages of the normalized relative transcript quantities from each endogenous control were further normalized to the first sample in the data set for each gene, so that the relative transcript quantity for each gene is equal to 1 for the first sample (i.e., B1F1).

### Statistical analysis

All analyses were done with three biological replications (five plants were collected for one biological replication). Gene transcript relative quantities were Z-transformed for all analyses to normalize the numerical range of relative transcript abundance between genes. Univariate analysis of variance (ANOVA) and Duncan’s multiple range test were performed using SPSS (Armonk, NY, USA). Principle component analysis (PCA) and Pearson’s correlation analysis was conducted by JMP Pro 12 (SAS Institute, Cary, NC, USA). The heatmap was generated by using MetaboAnalyst 3.0 (http://www.metaboanalyst.ca/faces/home.xhtml): Euclidean distance measure and Ward clustering algorithm were chosen for data analysis from normalized data with autoscale features as standardization. The top 25 most differentially expressed genes were selected based on T-test/ANOVA method for both broccoli and collard data sets. Tukey’s honest significant difference (HSD) test in MetaboAnalyst was used to examine significance of gene expression differences among tissues. Partial least square discriminant analysis (PLS-DA) in MetaboAnalyst was used to calculate variable importance in projection (VIP) value.

## Results and discussion

### Glucosinolate composition from various tissues of broccoli and collard

**Fig 1:**
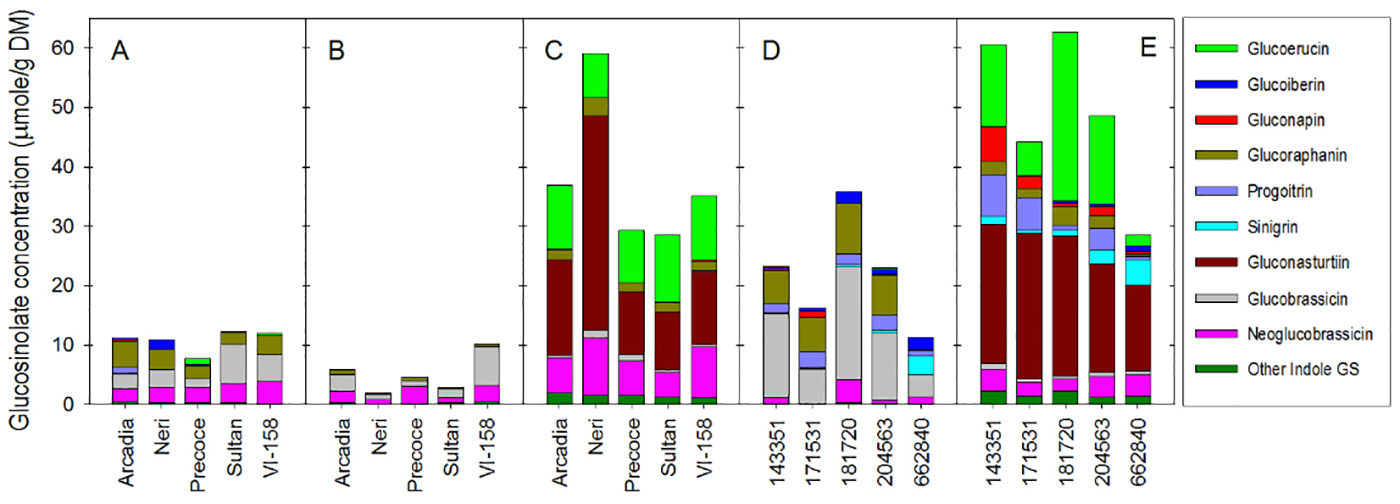
Glucosinolate profiles in extracts of floret (A), leaf (B), and root (C) tissue of broccoli and in leaf (D) and root (E) tissue of collards.

All five tested broccoli varieties showed similar tissue-dependent glucosinolate content differences, and mean total glucosinolate concentrations in root, floret, and leaf tissues of all tested five varieties were 37.8 μmol/g DW, 10.8 μmol/g DW, and 5.20 μmol/g DW, respectively (Fig 1A, 1B and 1C). There were variety-dependent variations in total glucosinolate concentrations in floret (ranged from 7.71 to 12.3 μmol/g DW), leaf (1.98 to 10.2 μmol/g DW), and root (28.5 to 59.1 μmol/g DW) tissues. Collards also showed tissue-dependent variation of total glucosinolate concentrations in all five tested varieties; the mean total glucosinolate concentration in collard roots was 48.9 μmol/g DW, which was over two-fold higher than that of collard leaf tissue (22.3 μmol/g DW) (Fig 1C and 1D).

In addition to differences between tissues for total glucosinolates in both broccoli and collards, there was also notable variation for the type of glucosinolates found in the different tissues assayed. In the case of broccoli, the major glucosinolates in florets were glucobrassicin (3.59 μmol/g DW, 33.2% of total glucosinolate), glucoraphanin (3.05 μmol/g DW, 28.2% of total glucosinolate), and neoglucobrassicin (2.84 μmol/g DW, 26.3%), while glucobrassicin (2.50 μmol/g DW, 48.6%) and neoglucobrassicin (1.81 μmol/g DW, 35.1%) were the major constituents in leaf tissue. In roots, however, gluconasturtiin (16.93 μmol/g DW, 44.8%) and glucoerucin (9.79 μmol/g DW, 25.9%), which consisted of less than 1% of total glucosinolates in floret and leaf tissues, were the dominant forms. Previous research indicates that root tissues of several selected *Brassica* crops have significantly higher levels of certain glucosinolates, including glucoerucin and gluconasturtiin, than leaves of the same crops [25].

In the case of collards, the total glucosinolate concentration in leaf and root tissues of the five tested accessions ranged 11.4 to 36.4 μmol/g DW and 28.6 to 62.6 μmol/g DW with average values of 22.3 μmol/g DW and 48.9 μmol/g DW, respectively. Similar to broccoli, collards also showed tissue-dependent differences in glucosinolate profiles in that glucobrassicin (10.9 μmol/g DW, 48.3% of total glucosinolate) and glucoraphanin (5.38 μmol/g DW, 24.1%) were the major glucosinolates in collard leaves, while gluconasturtiin (20.8 μmol/g DW, 42.6%) and glucoerucin (12.9 μmol/g DW, 26.3%) consisted of about 70% of total glucosinolates in collard roots.

**Fig 2:**
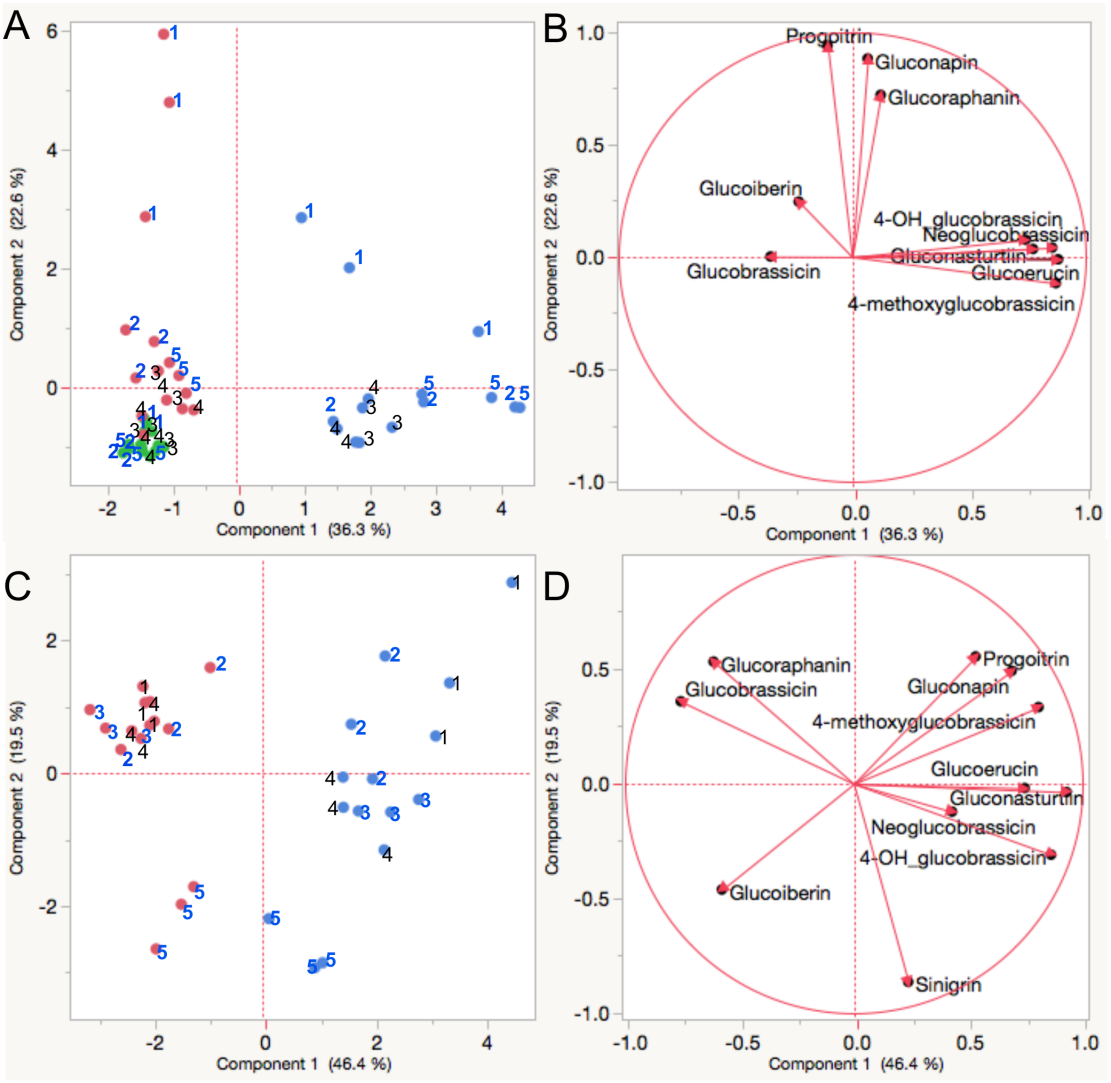
PCA analysis of glucosinolates from different tissues. Score(A) and loading (B) plot of various broccoli tissues: Different colors indicate various broccoli tissues (red: leaf; green: floret; blue: root) from tested varieties (1: ‘Arcadia’; 2: ‘Neri’; 3: ‘Precoce’; 4: ‘Sultan’; 5: ‘VI-158’). Score (C) and loading (D) plot of collard tissues: Different colors indicate various collard tissues (red: leaf; blue: root) from tested varieties (1: ‘PI143351’; 2: ‘PI171531’; 3: ‘PI181720’; 4: ‘PI204563’; 5: ‘PI662840’). Numbers in bold blue in figure (broccoli 1, 3, and 5 variety and collard 2, 3, and 5) were selected for targeted transcriptomics.

Glucosinolate composition of leaves and florets from the broccoli accessions were not distinctly different (Fig 2A). However, both broccoli and collards showed significantly different glucosinolate composition between aerial tissue (leaves and/or florets) and underground tissues (roots) (Fig 2A and 2C). A PCA score plot (Fig 2A) revealed that component 1 contributed to separation between aerial tissue and underground tissues for both broccoli (36.3%) and collard (46.3%). The PCA loading plot (Fig 2B) showed glucoerucin, gluconasturtiin, and several indole glucosinolates (4-methoxyglucobrassicin, 4-hydroxyglucobrassicin, and neoglucobrassicin) were positively associated with PCA component 1 in broccoli. These compounds also showed a similar association with PCA component 1 in collards (Fig 2D). Among tested broccoli varieties, ‘VI-158’ exhibited distinctive glucosinolate profiles in leaf and root tissues, where leaf tissues contained greater levels of glucobrassicin (6.53 μmol/g DW) and root tissues contained higher levels of gluconasturtiin (36.1 μmol/g DW) compared to the other varieties. ‘Arcadia’ broccoli showed the highest glucoraphanin concentration in florets; ‘Neri’ broccoli had the highest glucoiberin concentration in florets and lowest total glucosinolate concentration in leaf tissue.

Among the five tested collard accessions, the glucosinolate profile of collard accession ‘PI662840’ was unique because it contained the highest concentration of sinigrin (27.3% of total glucosinolates) and lowest glucoraphanin (1.0% of total glucosinolates) in leaf tissue, compared to other accessions. The root of ‘PI662840 displayed a comparatively high composition of sinigrin (14.7% of total glucosinolates) and low glucoerucin (6.4% of total glucosinolates) compared to other accessions. Collard accession ‘PI181720’ had significantly higher glucoerucin content in root tissue than other accessions. This accession also had the highest glucoraphanin concentration in leaf tissue. Thus, it was distinct in its PCA score (Fig 2C).

Based upon their unique glucosinolate profiles (Fig 1) and PCA analysis results, three varieties of broccoli (‘Neri’, ‘Arcadia’, and ‘VI-158’) and three accessions of collards (‘PI171531’, ‘PI181720’, and ‘PI662840’) were selected for transcriptomic analysis.

### Glucosinolate hydrolysis product content

**Fig 3:**
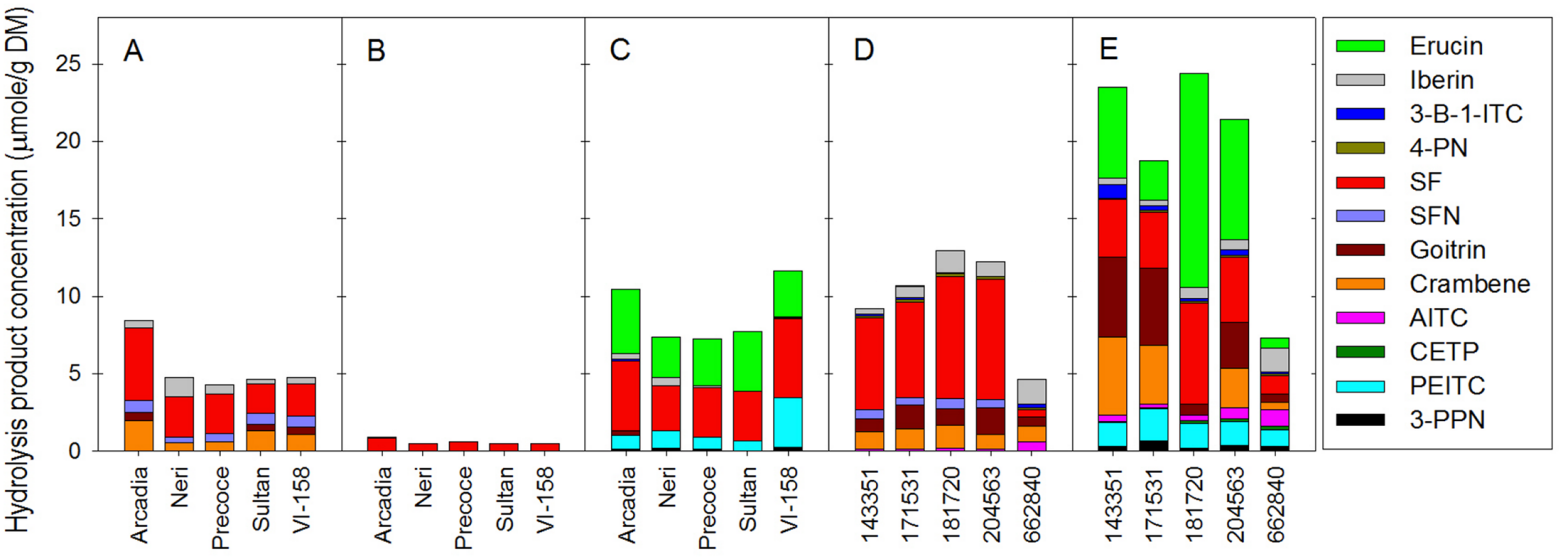
Glucosinolate hydrolysis product concentrations of floret (A), leaf (B), and root (C) tissue of broccoli and in leaf (D) and root (E) tissue of collards. 3-B-1-ITC stands for 3-buten-1-yl Isothiocyanate; 4-PN stands for 4-pentenenitrile; SF stands for sulforaphane; SFN stands for sulforaphane nitrile; AITC stands for allyl isothiocyanate; CETP stands for 1-cyano-2,3-epithiopropane; PEITC stands for 2-phenethyl isothiocyanate; 3-PPN stands for 3-phenylpropionitrile.

As to be expected with the observed differences in glucosinolate profiles, extracts from different tissues of broccoli and collards also showed different profiles and concentrations of glucosinolate hydrolysis products (HPs; Fig 3). In the case of broccoli, the average total HP concentration in root tissue extracts (8.88 μmol/g DW) was higher than in those from florets (5.38 μmol/g DW) and leaves (0.61 μmol/g DW). The primary forms of HPs in broccoli floret tissues were SF and crambene, which consisted of 51.3% and 20.6% of total HPs, respectively. SF was the major HP in broccoli leaf and root tissue extracts (96.3% and 42.9%, respectively). Erucin (37.1%) and PEITC (14.8%) were major HPs in broccoli root tissue extracts, which differentiated roots from other tissues.

In the case of collards, the average total hydrolysis product content in root tissue (19.08 μmol/g DW) was much higher than in leaves (9.93 μmol/g DW), the consumed portion of collard greens. On average, the major HPs in collard leaf tissue were SF (56.8%), goitrin (11.7%), crambene (11.7%), and iberin (10.1%), while erucin (32.2%), SF (20.2%), goitrin (15.1%), and crambene (12.4%) were major HPs in collard root tissue.

For both broccoli and collards, relatively small amounts of PEITC (1.32 and 1.56 μmol/g DW,^,^ respectively) were detected in root tissues compared to its precursor, gluconasturtiin (16.9 and 20.8 μmol/g DW, respectively) in the same tissues. This discrepancy is probably due to its lower solubility in water compared to other compounds, which has been previously reported [19, 26]. Although SF has a water solubility of 8.0 mg/mL, PEITC has only 0.051 mg/mL of water solubility [27]. For by-product utilization, lack of water solubility of PEITC should be considered as an important factor. The primary glucosinolate HPs in broccoli and collard roots were erucin and PEITC. These volatile hydrolysis products in broccoli and collard roots may be associated with plant-pathogen soil interactions. A recent publication has reported that AITC altered soil microbial community composition [28]. This suggests that non-synthetic anti-microbial agents can be developed from broccoli and collards root materials.

For SF, conversion rate from glucoraphanin averaged 108% from floret and leaf tissues in broccoli and collard (n = 13, two very high outlying conversion rates were excluded due to very low glucoraphanin concentrations). Surprisingly conversion of glucoraphanin to SF was 213% from broccoli and collard roots. This is likely due to to the reported conversion of erucin to SF following hydrolysis [29]. The conversion rate of glucoraphanin to SF (SF concentration / glucoraphanin concentration) was significantly correlated with glucoerucin concentration in root tissues from both broccoli and collard (r = 0.691, p = 0.027, n = 10). Previously, conversion of SF to erucin was reported as S-oxide reduction by reductase in gut bacteria [29, 30]. However, the interconversion mechanism from erucin to SF is currently unknown.

Nitrile formation percentage was significantly different in the various broccoli tissues assayed (S3 Table). Floret tissues had an average of 30.6% of nitrile forms among all HPs, while leaf and root tissues had a significantly lower nitrile formation percentage (0 and 2.2%, respectively). Collard leaves and root tissues averaged 19.5% and 14% nitrile formation, respectively. It has been reported that broccoli florets have considerable ESP activity, which is associated with enhanced nitrile formation [15].

### Quinone reductase activity

**Fig 4:**
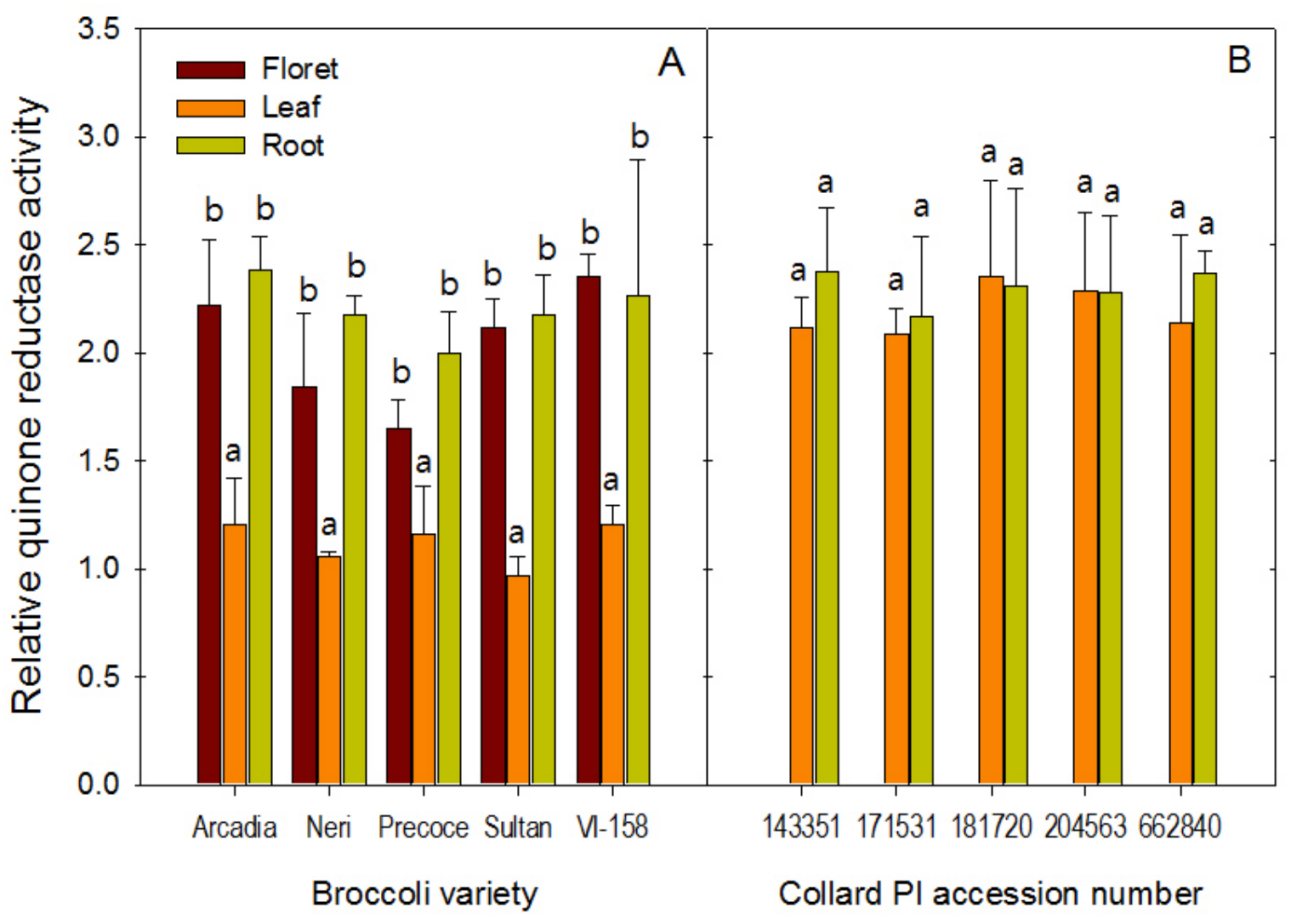
Relative quinone reductase-inducing activity of floret, leaf, and root extracts of broccoli (A) and collards (B). Vertical bars represent mean and standard deviation of 3 independent biological replications. Different letters within a variety represent a statistically significant difference at p<0.05 by Duncan's multiple range test.

Unlike the prominent differences in glucosinolate and their HP concentrations, the QR inducing effects exhibited significant variation only between plant tissues, not between accessions. Extracts from root tissues of broccoli and collards were not significantly different from the QR inducing activity of broccoli florets and collard leaves, respectively (Fig 4). Relatively low QR inducing activity of root samples despite of high glucosinolate and HP concentration could be explained by volatility of HPs in root samples. As mentioned previously, PEITC and erucin have very low water solubility, which has been previously reported [19, 26]. Previous studies could not detect PEITC in hydrolyzed watercress extracts due to water solubility [31]. Thus, only trace amounts of these compounds were likely dissolved in water hydrolysis extracts used for the QR assay, even though PEITC and erucin were well-extracted by conducting liquid to liquid solvent extraction for hydrolysis product analysis (Fig 3). This difference between QR inducing ability and glucosinolate/HP profile has been reported previously [31, 32].

To the best of our knowledge, this is the first report of bioactivity from broccoli and collard roots, since they are not typically consumed plant tissues. QR inducing activities of broccoli floret extracts were significantly higher than broccoli leaf extracts of all correspondant cultivars/accessions. QR inducing activity of collard leaf tissues was much higher than broccoli leaf tissues, even though they are both *Brassica oleracea*. The higher concentration of SF in collard leaf tissue may explain this difference in this QR inducing activity. Possibly, the different developmental stages of broccoli (reproductive stage) and collards (vegetative stage) when harvested affect glucosinolate accumulation in leaf tissues. Long-distance phloem transport of glucosinolates has been shown to occur in *Arabidopsis* [33]. No data on glucosinolate accumulation in broccoli leaf tissue at various development stages could be found at this time, and therefore, more research is required to confirm this hypothesis.

Significant correlations were found between precursor glucosinolates and their hydrolysis products (S4 Table). There were significant correlations between QR inducing activity and several phytochemicals, including: total aliphatic glucosinolates, r = 0.581, p = 0.023, n = 15; total glucosinolates, r = 0.592, p = 0.020, n = 15; SF, r = 0.597, p = 0.019, n = 15; and total isothiocyanates, r = 0.545, p = 0.017, n = 15. Of the glucosinolate HPs, SF is often reported to have the highest induction capability for Phase II antioxidant enzymes, including QR [8]. However, Phase II induction has also been described for other isothiocyanate HPs (reviewed by Becker and Juvik, 2016), lending credence to the almost equally strong correlation between QR and total isothiocyanates seen in our results.

### Targeted transcriptomics of glucosinolate biosynthesis and hydrolysis

**Fig 5:**
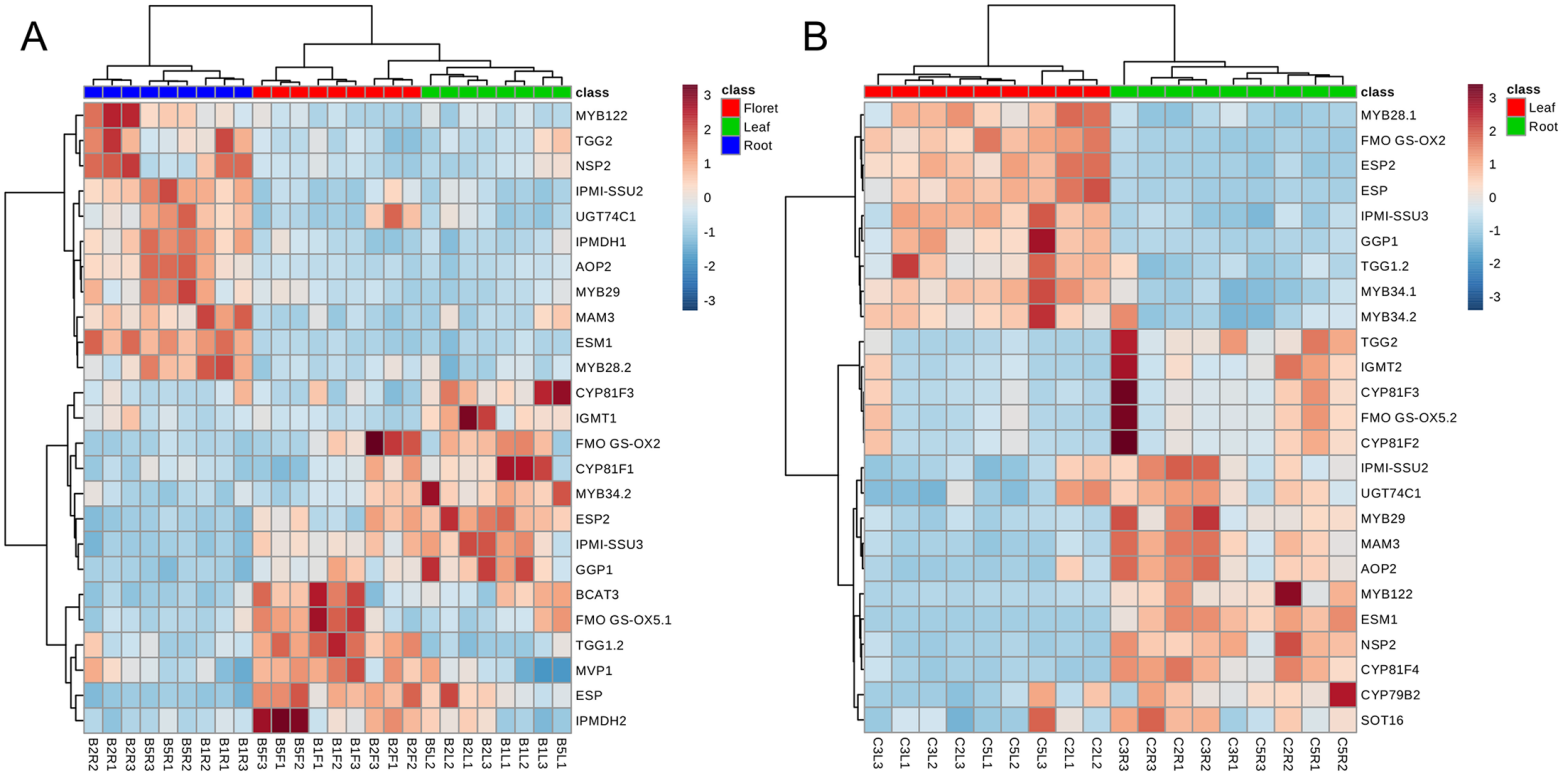
Heatmap of gene expression from various broccoli (A) and collard (B) tissues. The top 25 most differentially expressed genes were selected. B1: ‘Arcadia’ broccoli; B3: ‘Broccoli Neri (PI662531)’ broccoli; B5: ‘VI-158’ broccoli. C2: ‘PI171531’ collard; C3: ‘PI181720’ collard; C6: ‘PI662840’ collard.

In an effort to better understand the transcriptional control of the glucosinolate biosynthetic mechanism, three broccoli and three collard cultivars were selected on the basis of their comparatively unique glucosinolate profiles and PCA results (Figs 1 and 2). From the heatmap (Fig 5A), gene expression patterns of each tissue type were distinct. Transcript abundance of *ESM1*, *IPMI-SSU2*, *IPMDH1*, *AOP2*, *MAM3*, *MYB28*.*2*, *MYB122*, *MYB29*, *TGG2*, *NSP2*, *and UGT74C1* was significantly higher in root tissue than aerial tissues, while gene expression levels of *ESP2*, *ESP*, *IPMI-SSU3*, *GGP1*, *FMOGS-OX2*, and *BCAT3* from root tissue were significantly lower than aerial tissues (by Tukey’s HSD at 0.01; S5 Table). In addition, a leaf-specific higher gene expression pattern was observed for *MYB34*.*2 CYP81F3*, *IGMT1*, *CYP81F1* compared to other tissues (by Tukey’s HSD at 0.01; S5 Table). *TGG1*.*2*, *IPMDH2*, and *MVP1* showed significantly higher gene expression in floret tissue compared to the other two tissues, while *FMOGS-OX2* transcript was significantly more abundant in root tissue (by Tukey’s HSD at 0.01; S5 Table).

The gene expression data help to explain why roots contained high glucoerucin concentrations compared to leaves and florets, since these tissues displayed significantly lower expression levels of *FMO GS-OX2* and *FMO GS-OX5*.*1*, genes which are responsible for the synthesis of glucoraphanin from glucoerucin (S5 Table). Moreover, high *AOP2* gene expression explains relatively lower glucoraphanin concentration than glucoerucin in root (Fig 5A). Broccoli florets usually have high glucoraphanin levels, which indicates that *FMOGS-OX* and *AOP2* are usually expressed at high and low levels, respectively, for the accumulation of glucoraphanin. The redundant homologs of *FMOGS-OX* were complementarily expressed in broccoli florets dependent on the broccoli variety. For example, ‘Arcadia’ and’ VI-158’ had high expression of *FMOGS-OX5*.*1* rather than *FMOGS-OX2*, while ‘Neri’ and all three broccoli leaf tissues surveyed show the opposite pattern (Fig 5A). Higher gene expression levels of *ESP* in broccoli florets and *ESP2* in broccoli leaf tissues with lower levels of *ESM1* in leaf and floret compared to root tissues may explain the observed high nitrile formation percentage (S3 Table). The gene expression mean value of *ESM1* in root tissue was 104 and 25-fold higher than floret and leaf tissues, respectively (calculated from relative quantification). The gene expression of *ESP* in root was 16-fold lower than floret tissue (calculated from relative quantification). There was a weak correlation between *ESP* gene expression and nitrile formation percentage (r = 0.490, p = 0.065, n = 15). However, there was significant correlation between *ESM1* and *ESP* (r = -0.873, p<0.0001, n = 15) as well as between *ESM1* and *ESP2* (r = -0.806, p = 0.0003, n = 15). The weak correlation between nitrile formation and gene expression of *ESP* may be explained by the fact that *ESM1* regulates *ESP* activity [11, 14]. Broccoli and collard roots appear to be an excellent source of active myrosinase without high ESP activity, ensuring high rates of SF formation from available glucoraphanin.

These two genes were the top two variables of importance in projection (VIP) from the partial least square discriminant analysis (PLS-DA, S6 Table). The VIP values of *ESM1* and *ESP* were 1.87 and 1.71, respectively. VIP values greater than 0.8 make an important contribution to the dimensionality reduction involved in PLS compared to variables less than 0.8 [34]. Fourteen variables had a VIP value higher than 1.2 for broccoli tissue transcript abundance profiles (S6 Table). These could be useful transcript level biomarkers to indicate different broccoli tissues. Certain paralogous genes were expressed differently in various tissues (i.e, *TGG1*.*2* and *TGG2*; *IPMI-SSU2* and *IPMI-SSU3*; *IPMDH1* and *IPMDH2*). As previously discussed, *TGG1* and *TGG2* have redundant function in glucosinolate hydrolysis and insect defense [35]. *TGG2* seems highly expressed in broccoli root compared to *TGG1*.*2*.

In the case of collards, similarly, transcript abundance of *MYB28*.*1*, *FMO GS-OX2*, *ESP2*, *ESP*, *IPMI-SSU3*, *GGP1*, *TGG1*.*2*, *MYB34*.*1*, and *MYB34*.*2* in collard root tissues were significantly lower compared to leaf tissue (by T-test at α = 0.01; S7 Table). Gene expression of *TGG2*, *FMOGS-OX5*.*2*, *CYP81F4*, *IPMI-SSU2*, *MYB29*, *MAM3*, *AOP2*, *MYB122*, *ESM1*, *NSP2*, and *CYP81F4* in collard roots showed significantly greater transcript abundance compared to leaf tissue (by T-test at α = 0.01; S7 Table). To a slightly lesser extent, gene expression of *IGMT2*, *CYP81F3*, *UGT74C1*, *FMOGS-OX5*.*2* was also significantly greater in collard root tissue compared to leaf tissue (by T-test at α = 0.05; S7 Table). Although *FMOGS-OX5*.*2* was relatively highly expressed in root tissue, *FMOGS-OX2* in roots was suppressed compared to leaf tissue (Fig 5B). This suggests that this may be the limiting factor in glucoerucin accumulation in collards. Unlike broccoli, *FMOGS-OX5*.*2* may be a non-functional gene or not a contributing factor in the conversion of glucoerucin to glucoraphanin. VIP values of *ESM1* and *FMOGS-OX2* were 1.57 and 1.56, making them the top two genes, followed by *ESP2* (VIP = 1.53). 16 variables in the collards data generated VIP values greater than 1.2 (S6 Table). Similar to the broccoli samples, certain paralogous genes were expressed differently in various tissues (i.e, *TGG1*.*2* and *TGG2*; *IPMI-SSU2* and *IPMI-SSU3*; *FMOGS-OX2* and *FMOGS-OX5*.*2*).

Gene expression of *ESP2* was negatively correlated with QR activity (r = -0.829, p<0.0001, n = 15) while gene expression of *ESM1* was positively correlated with QR activity (r = 0.551, p = 0.003, n = 15) (S4 Table). Total isothiocyanate concentration was positively correlated with the transcript abundance of glucosinolate side-chain elongation genes (*MAM3*, *IPMDH1*, *BCAT4*, and *IPMI-SSU2*), core structure formation genes (*UGT74C1*), transcription factors associated with aliphatic glucosinolate biosynthesis (*MYB28*.*2* and *MYB29*), side chain modification genes (*AOP2*), and the myrosinase cofactor, *ESM1*. Total isothiocyanate concentration was negatively correlated with *ESP2* (r = 0.607, p = 0.016, n = 15). Previously, it was reported that increased expression of *MYB28* homologs achieved by the introgression of chromosomal segments from *Brassica vilosa* (a wild *Brassica* species) led to high accumulation of glucoraphanin in ‘Beneforte’, a recently released broccoli hybrid [36].

**Fig 6:**
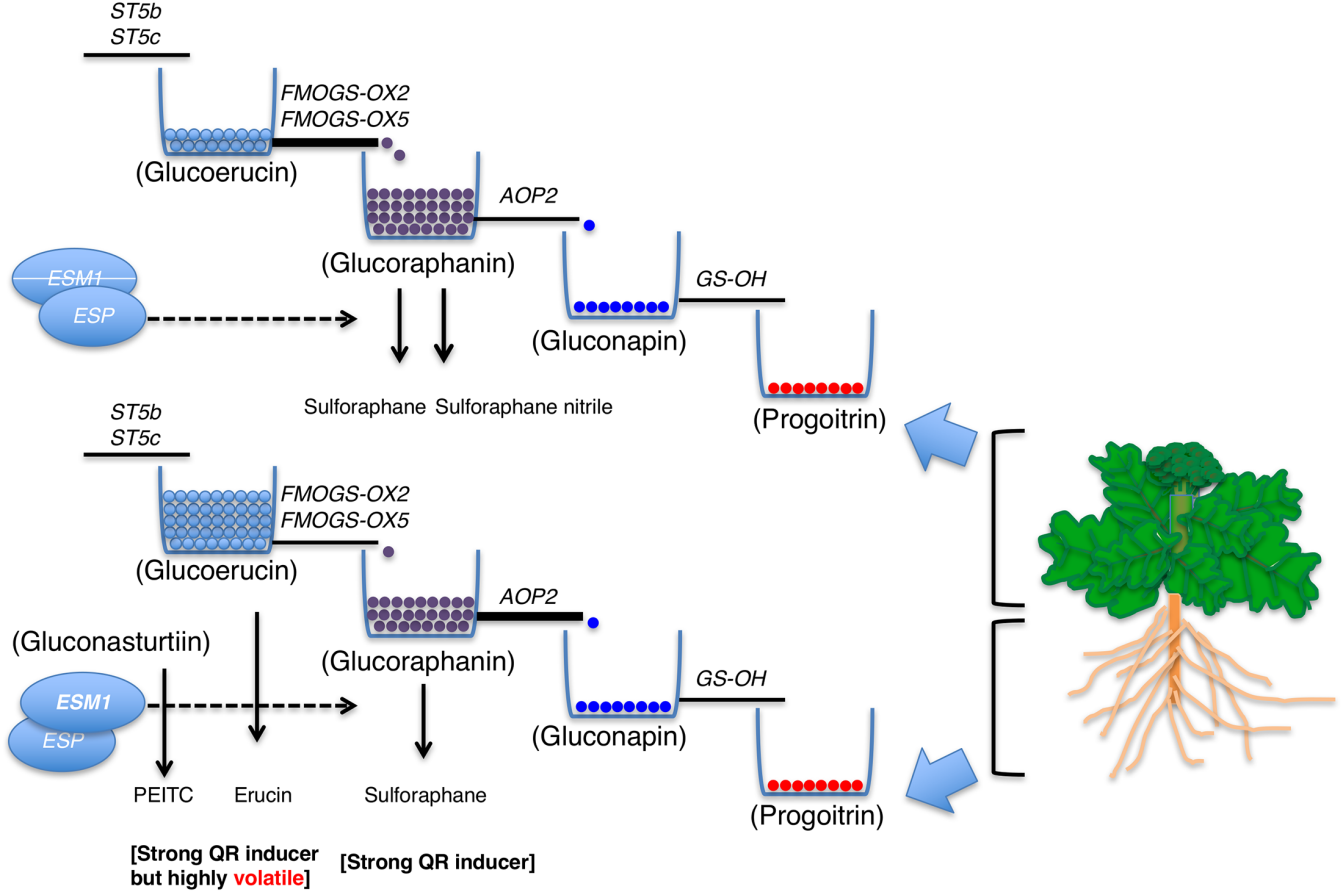
Proposed gene regulation of glucoraphanin biosynthesis in various tissues of broccoli and quinone reductase inducing activity. Upper part indicates glucosinolate biosynthesis in broccoli florets and leaves. Bottom part indicates glucosinolate biosynthesis in broccoli roots. Each reservoir indicates pool of glucosinolate concentration. Size of each pipeline attached to reservoir indicates expression level of each gene. Accumulated glucoraphanin concentration is associated with limited expression of the *AOP2* gene (narrow pipeline) and elevated expression of *FMOGS-OX2*, *5* (thick pipeline). High concentrations of glucoraphanin in broccoli florets contribute to quinone reductase (QR) inducing anticancer activity. However, roots have shown high expression of *ESM1* and low expression of *ESP* homologs, resulting in a high percentage of isothiocyanate over nitrile form, which contributes to QR inducing activity in root tissues. Elevated concentrations of gluconasturtiin and glucoerucin likely did not contribute to quinone reductase inducing anticancer activity because of high hydrolysis product volatility.

For breeding purposes, high accumulation of glucoraphanin requires high expression of *FMOGS-OX* and low expression of *AOP2* (Fig 6). For high glucoraphanin conversion to SF, high expression of *ESM1* and low expression of *ESP* homologs is required. Thus, these genes should be considered as important biomarkers. In a previous study, RNAi knockout in *Brassica juncea* of *AOP2*, responsible for the conversion of glucoraphanin to other downstream glucosinolates, resulted in an increase in the concentration of glucoraphanin from approximately 0 μmole/g to greater than 40 μmole/gram in one transformant [37]. Thus, comparison among various tissues that have distinct glucosinolate and transcriptional profiles may provide insight into how we may best be able to manipulate glucosinolate biosynthesis to produce valuable compounds in specific plant tissues.

Using the Fluidigm system, we were able to measure gene expression for our selected target gene set (n = 45, without references genes) for 44 samples within 2 days with minimized human pipette error by utilizing microfluidic reaction. Note that a few sample spots were used for establishing a relative calibration curve for quantification. The Fluidigm system allows us to measure gene expression for a selected gene set (up to 96) and samples (up to 96) with far greater ease and speed compared to conventional RT-qPCR with 384 well microtiter plates. This system allows for a 50–100 fold reduction in the amount of material/reagents consumed, with 5 to 20 fold increased throughput compared to conventional single-plex RT-qPCR [38]. Therefore, this system could be an excellent tool for scientists to conduct targeted transcriptomics research.

Given our results, broccoli and collard roots can be good sources for glucosinolates with favorable molecular conditions for isothiocyanate production during hydrolysis by endogenous myrosinase, if desired (very low ESP activity and high *ESM1* expression). There has been some research aimed at producing glucosinolates using a hairy root culture system with broccoli and *Arabidopsis* [39, 40]. A broccoli hairy root culture system with supplemented auxins has been shown to be more effective to produce indole glucosinolates compared Murashige and Skoog medium [41]. These methods may be a more feasible practice to utilize root tissue for bioactive glucosinolate production. However, research using the broccoli hairy root culture system has focused on indole glucosinolate production, although we have shown broccoli roots to have high concentrations of gluconasturtiin and glucoerucin. Modifying the broccoli hairy root culture system to include production of these aromatic and aliphatic glucosinolates requires more research. Harvesting broccoli or collard roots for bioactive compound extraction may not be feasible due to harvest and labor costs. Broccoli and collard roots have many small or hairy roots, meaning only a portion of the root biomass is available for harvesting in the form of the taproot. Considering the harvesting and transporting of these by-products also requires labor and efforts, by-product utilization for on-site control of soil-born disease may be feasible utilization. In a previous study, broccoli residue incorporation into the soil effectively reduced Verticillium wilt incidence on cauliflower and eggplant [42, 43]. In order to utilize glucosinolates from root tissues for bioactive compound production, additional economic and logistic based research is needed to determine feasibility.

## Conclusions

This study has found that root tissues have a high concentration of gluconasturtiin and glucoerucin, and their isothiocyanate HPs, compared to other tissues in broccoli and collards. Low nitrile formation capacity in root tissues, possibly related to the high observed *ESM1* and low *ESP* gene transcription, make the roots of broccoli and collards, and perhaps all *Brassica oleracea* crops, a good source of active myrosinase and precursor glucosinolates of bioactive HPs. In addition, comparative analysis reveals the metabolomic and transcriptomic differences among various tissues and between different *Brassica oleracea* crops. This information may be helpful for the utilization of by-products of broccoli and collards, either for human health, pest management, or anti-pathogen agents. In addition, the mechanism of glucoraphanin biosynthesis and hydrolysis in broccoli florets was generally associated with transcript abundance, providing us with insight on how to best manipulate glucosinolate levels for any number of possible breeding goals.

## Supporting information

S1 Table GC-MS spectra of hydrolysis products of glucosinolates. (DOCX)

S2 Table List of primers used for transcript abundance profiling (RT-qPCR). (DOCX)

S3 Table Total nitrile formation (%) from various broccoli and collard tissues. (XLSX)

S4 Table Correlations among glucosinolate, glucosinoalte HP concentrations, and transcript. (XLSX)

S5 Table ANOVA posthoc result with Tukey’s HSD test from broccoli gene expression data. (XLSX)

S6 Table VIP value from PLS-DA. (XLSX)

S7 Table T-test result from collard gene expression data. (XLSX)

S1 Fig Typical HPLC chromatogram of glucosinolates in broccoli ‘Arcadia’ floret (A), leaf (B), and root (C). (TIF)

S2 Fig Typical chromatograms of glucosinolates in collard (Acc. 662840) leaf (A) and root (B). (TIF)

S3 Fig Typical chromatogram of glucosinolate hydrolysis products in broccoli ‘VI-158’ floret (A), leaf (B) and root (C). (TIF)

S4 Fig Typical chromatogram of glucosinolate hydrolysis products in collard (Acc. 662840) leaf (A) and root (B). (TIF)
