# Supplementary material for: Chemopreventive glucosinolate accumulation in various broccoli and collard tissues: Microfluidic-based targeted transcriptomics for by-product valorization
Source: PLoS One. 2017 Sep 25;12(9):e0185112. doi: 10.1371/journal.pone.0185112 (PMC5612653; doi:10.1371/journal.pone.0185112)
Supplement: S1 Table — (DOCX) [file pone.0185112.s001.docx]

**Supplementary S1 Table**. GS-MS spectra from glucosinolates hydrolysis standards. Sulforaphane nitrile mass spectrum was obtained by broccoli floret tissue.

| Chemical name | Formula  /M.W. | Mass spectra |
| --- | --- | --- |
| Erucin | C_6_H_11_NS_2_  161.28 | 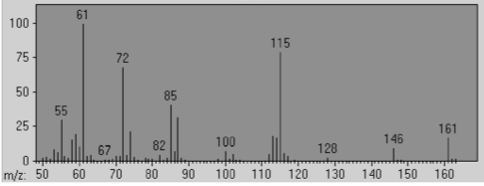 |
| Iberin | C_5_H_9_NOS_2_ 163.25 | 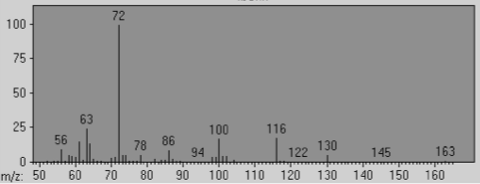 |
| 3-butenyl isothiocyanate | C_5_H_7_NS  113.18 | 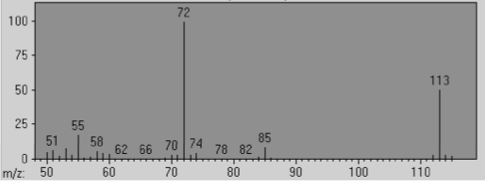 |
| 4-pentenyl isothiocyanate | C_6_H_9_NS  127.21 | 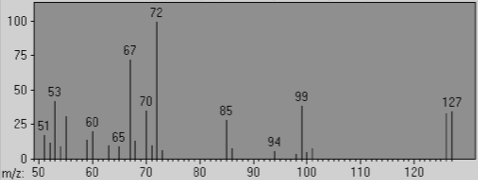 |
| Sulforaphane | C_6_H_11_NOS_2_  177.29 | 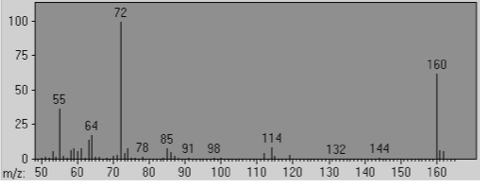 |
| Suforaphane nitrile | C_6_H_11_NOS  145.22 | 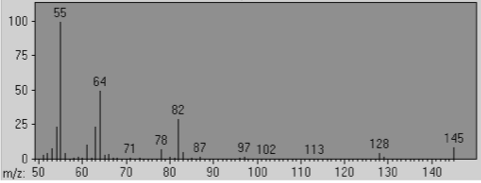 |
| Goitrin | C_5_H_7_NOS  129.182 | 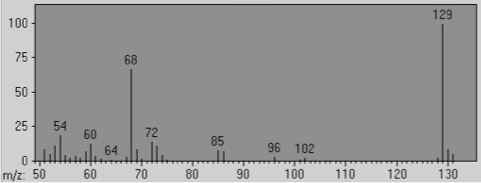 |
| Crambene  (1-Cyano-2-hydroxy-3-butene)^z^ | C_5_H_7_NO  97.12 | 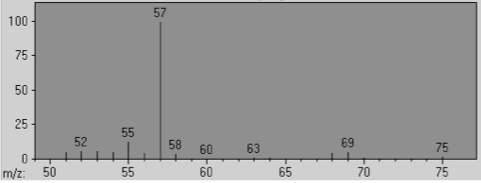 |
| Allyl isothiocyanate | C_4_H_5_NS  99.15 | 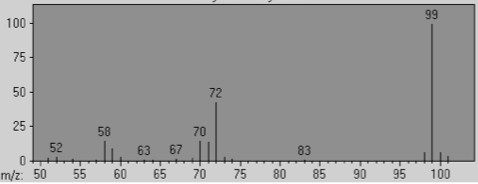 |
| 1-Cyano-2,3- epithiopropane | C_4_H_5_NS  99.15 | 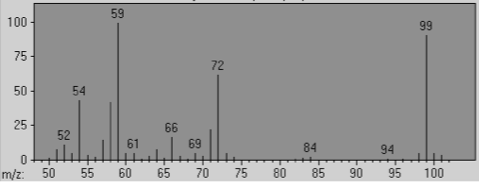 |
| 2-phenethyl isothiocyanate | C_9_H_9_NS  163.24 | 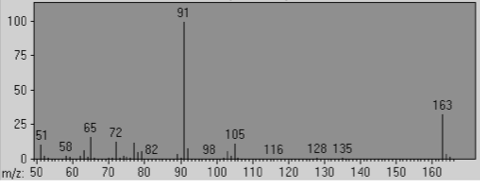 |
| 3-phenylpropionitrile | C_9_H_9_N  131.17 | 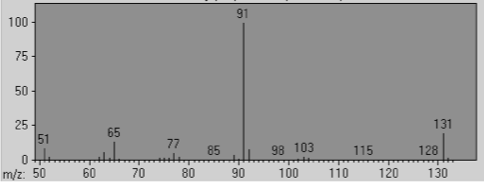 |
