# Supplementary material for: Chemopreventive glucosinolate accumulation in various broccoli and collard tissues: Microfluidic-based targeted transcriptomics for by-product valorization
Source: PLoS One. 2017 Sep 25;12(9):e0185112. doi: 10.1371/journal.pone.0185112 (PMC5612653; doi:10.1371/journal.pone.0185112)
Supplement: S2 Table — (DOCX) [file pone.0185112.s002.docx]

Table S2. List of primers used for transcript abundance profiling (RT-qPCR).

| **Gene Symbol** | **Primer Name**^a^ | **Sequence** | **Start bp** | **Stop bp** | **Tm** | **Avg. Tm** | **Amplicon (bp)** |
| --- | --- | --- | --- | --- | --- | --- | --- |
| Actin 2 | Bol030974 F | TCCCGAGAGGAAGTACAGTGTCT | 1002 | 1024 | 58 | 58 | 77 |
|  | Bol030974 R | GAGATCCACATCTGCTGGAATG | 1079 | 1058 | 58 |  |  |
| TBP1 | Bol040381 F | TGTGGCGATCGAGAGCAAT | 72 | 90 | 59 | 58.5 | 67 |
|  | Bol040381 R | GAATGGCGCAGAAAAGAAGAA | 139 | 119 | 58 |  |  |
| SAND1 | Bol013385 F | TGAACTCTACGCAGCATTTGATC | 1629 | 1652 | 58 | 58 | 71 |
|  | Bol013385 R | TGGCACACCTGATTGCATATC | 1700 | 1721 | 58 |  |  |
| MYB 28.1 | Bol036286 F | TCTGAGCAGATTCTCAATGAAGATG | 241 | 265 | 59 | 58.5 | 97 |
|  | Bol036286 R | TCAGGGTAAAACGTTGTTTGGA | 338 | 317 | 58 |  |  |
| MYB 28.2 | Bol007795 F | CTCTTCCTCTTTCCTCGGGTTT | 14 | 35 | 64 | 65 | 74 |
|  | Bol007795 R | TGCAACTCAAGGAACCTCTCTGA | 88 | 66 | 66 |  |  |
| MYB 29 | Bol008849 F | GCTTCCATGGGCAATATCATATC | 151 | 173 | 58 | 58 | 75 |
|  | Bol008849 R | GACATGGAGGAGACAGTGTTGTAGA | 226 | 202 | 58 |  |  |
| MYB34.1 | Bol017062v3 F | TGAAGGTGGATGGCGTACTCT | 99 | 120 | 58 | 58 | 96 |
|  | Bol017062v3 R | GCCCATCTCAGCCTACAACTCT | 195 | 173 | 58 |  |  |
| MYB34.2 | Bol007760 F | GCTCAAACCGGTGGCAAA | 582 | 600 | 59 | 58.5 | 89 |
|  | Bol007760 R | CGTCAAGATCATCGGAGAAAGA | 671 | 649 | 58 |  |  |
| MYB122 | Bol026204 F | CTTCCCGACAAAGCTGGACT | 155 | 175 | 58 | 58 | 98 |
|  | Bol026204 R | TTGGCTAAACTCACCACGCT | 253 | 223 | 58 |  |  |
| BCAT4 | Bol018130 F | ACAGAAGATGGCCGGATTGT | 241 | 260 | 58 | 58.5 | 75 |
|  | Bol018130 R | AAAGTCTGTTGGCACCCGATT | 316 | 296 | 59 |  |  |
| MAM 1/2 | Bol037823 F | CTGGCATCTCCCCCAATG | 16 | 33 | 58 | 58.5 | 64 |
|  | Bol037823 R | CATGTTAAGCCTGATCGGACAA | 80 | 59 | 59 |  |  |
| IPMI-LSU1 | Bol040343 F | TGTGGTGCCTGCCTTGGT | 1396 | 1413 | 60 | 59.5 | 69 |
|  | Bol040343 R | TCGAGACACACACTTGAGGTTCA | 1465 | 1443 | 59 |  |  |
| IPMI-LSU2 | Bol029979 F | CAGCTGACAAAGCCACCATCT | 281 | 301 | 59 | 58.5 | 72 |
|  | Bol029979 R | CCGGAGAGAGCGTTGGAA | 353 | 336 | 58 |  |  |
| IPMI-SSU3 | Bol029441 F | CGAGCCGCAGGAGAGAAAG | 165 | 183 | 60 | 59.5 | 70 |
|  | Bol029441 R | TCTGGTCCGTGTCGATGTTG | 235 | 216 | 59 |  |  |
| IPMDH1 | Bol004236 F | CAATCTCGTTCCAGGCAGATC | 42 | 62 | 58 | 59 | 65 |
|  | Bol004236 R | GCGGCGCACCTTACTCTACA | 107 | 88 | 60 |  |  |
| IPMDH2 | Bol000668 F | GCTGGAGAGGATAAAGCAAACC | 985 | 1006 | 58 | 58.5 | 69 |
|  | Bol000668 R | CAAGCCCGTATTTCAGAAGCA | 1054 | 1034 | 59 |  |  |
| BCAT3 | Bol037342 F | CTCGGAACCTATCTCCGTCATC | 98 | 119 | 59 | 58.5 | 74 |
|  | Bol037342 R | GGGAGGTTCCGCCATTG | 172 | 156 | 58 |  |  |
| CYP79F1 | Bol038222 F | GGTCAAAGCTCAATGCGTTGA | 891 | 911 | 60 | 59.5 | 81 |
|  | Bol038222 R | CATTTCCGCAAGTGTCCATTC | 972 | 952 | 59 |  |  |
| UGT74C1 | Bol014127 F | CACCATCCACGACGGTTTCT | 177 | 196 | 59 | 58.5 | 66 |
|  | Bol014127 R | AAAACGTGGAGGGTCTTTGATC | 243 | 222 | 58 |  |  |
| SOT17 | Bol030757 F | CCATCGCCACGCTTCCT | 80 | 96 | 59 | 59 | 63 |
|  | Bol030757 R | CCGCCGTACTCGACGAAA | 143 | 126 | 59 |  |  |
| SOT18 | Bol026202v2 F | CCCAAAGACAGGCACCACTT | 255 | 274 | 59 | 58.5 | 72 |
|  | Bol026202v2 R | GGAATCGTCGAAGCGAGATC | 327 | 308 | 58 |  |  |
| CYP79B2 | Bol032767 F | GATGAAATTAAACCCACCATTAAGGA | 901 | 926 | 59 | 58.5 | 79 |
|  | Bol032767 R | GCCATGGCCCATTCGA | 980 | 965 | 58 |  |  |
| CYP79B3 | Bol031784v2 F | CCGTCGCCGCGTTTAAC | 1202 | 1218 | 60 | 59 | 82 |
|  | Bol031784v2 R | CACTTGACTTCCTTTAGGGATATGG | 1284 | 1260 | 58 |  |  |
| SOT16 | Bol039395 F | TTCGACGACGCCACGAA | 295 | 311 | 59 | 58.5 | 62 |
|  | Bol039395 R | CTCCACGTAAGGCACGAACTC | 357 | 337 | 58 |  |  |

^a^The gene model used for the creation of each primer set is indicated in the primer name.

Primer sets created from cDNA sequences not included in Liu et al. (2014) annotations that were chosen based on BLAST bit-score for similarity with A. thaliana annotated sequences are indicated in bold

Table S1 cont. List of primers used for transcript abundance profiling (RT-qPCR).

| **Gene Symbol** | **Primer Name^a^** | **Sequence** | **Start bp** | **Stop bp** | **Tm** | **Avg. Tm** | **Amplicon (bp)** |
| --- | --- | --- | --- | --- | --- | --- | --- |
| GGP1 | Bol018073 F | TTGACAAGTACGATGGTTTCGTTATT | 170 | 195 | 59 | 58.5 | 81 |
|  | Bol018073 R | TCACATAGCTTAAGGATCCAAGGAT | 251 | 227 | 58 |  |  |
| UGT74B1 | Bol005786 F | CGACGGCCACGACTTCAT | 114 | 131 | 59 | 59 | 70 |
|  | Bol005786 R | GCTTGAAGGATTCGGAGTATGC | 184 | 163 | 59 |  |  |
| FMO GS-OX2 | Bol010933v2 F | CCGGAGCATCTGGATTAATAGC | 53 | 74 | 59 | 59 | 82 |
|  | Bol010933v2 R | CACTTGTTTCTCCCGCTCAAA | 135 | 115 | 59 |  |  |
| FMO GS-OX5.1 | Bol031353v2 F | GAGCTTTGACTCCGCTATGGA | 729 | 749 | 58 | 58.5 | 77 |
|  | Bol031353v2 R | TTTACTTGTAGCGCACGTTTCG | 806 | 785 | 59 |  |  |
| FMO GS-OX5.2 | Bol029100 F | AGCTACGCCGCGAATCAC | 83 | 100 | 59 | 59 | 66 |
|  | Bol029100 R | ACCCAGAGACCTCCGATTTGT | 149 | 129 | 59 |  |  |
| AOP2 | AY044425.1 F | TGGGTGCAGACACTCCTCAA | 279 | 298 | 59 | 58.5 | 74 |
|  | AY044425.1 R | CCCACTTCTCACTTCCTGGTTT | 353 | 332 | 58 |  |  |
| GS-OH | Bol033373 F | GCTTGTTGATGCTCTGTCATTGT | 17 | 39 | 58 | 58.5 | 62 |
|  | Bol033373 R | TGGCGCCGAGCGTTAG | 79 | 64 | 59 |  |  |
| CYP81F1 | Bol028913 F | CCGAGACATTCCGGCTATTC | 1064 | 1083 | 58 | 58.5 | 64 |
|  | Bol028913 R | CATGTCCTCCGTCGGTCTTC | 1128 | 1109 | 59 |  |  |
| CYP81F4 | Bol032712v2 F | TCCCTCTCCGCCTCACTCT | 90 | 108 | 59 | 59.5 | 64 |
|  | Bol032712v2 R | GGTGGACGGGAGGTTTAATGA | 154 | 134 | 60 |  |  |
| IGMT1 | Bol007030 F | GGACCGGATGCTTCGTCTAC | 261 | 280 | 58 | 58.5 | 69 |
|  | Bol007030 R | TCTCTCGCCCTTTCCAAACTT | 330 | 310 | 59 |  |  |
| IGMT2 | Bol020661 F | CGCAGCCTTCCCAATGG | 105 | 121 | 60 | 59 | 68 |
|  | Bol020661 R | GCTTCGGCATAGAGAATGTCAA | 173 | 152 | 58 |  |  |
| TGG1.1 | Bol017328v2 F | GTGCCTACGAGAGGCTATTCAAC | 628 | 650 | 58 | 58 | 77 |
|  | Bol017328v2 R | GCCGTAACATCTTTCATCAACCT | 705 | 683 | 58 |  |  |
| TGG1.2 | Bol019343v2 F | AACCCCTTTTGTTACCCTCTATCA | 189 | 212 | 58 | 58 | 76 |
|  | Bol019343v2 R | TGCGGTTCAAGAAACCTTCAT | 265 | 245 | 58 |  |  |
| TGG2 | Bol028319v2 F | CGAACTCAACGCTACTGGTTACA | 342 | 364 | 58 | 58 | 78 |
|  | Bol028319v2 R | TACTCCCCTGCTCCTCTTTCC | 420 | 400 | 58 |  |  |
| PYK10 | Bol023070 F | GAAACATTGGATCACTTTCAACGA | 585 | 608 | 59 | 59 | 72 |
|  | Bol023070 R | TGGTGCTTTCTTGCCTACATCA | 657 | 636 | 59 |  |  |
| PEN2 | Bol030092 F | CGCTTTTCGTGAAGAGTATCAAAC | 614 | 637 | 58 | 58.5 | 78 |
|  | Bol030092 R | TTCATCCAATCTACGCCTTGATC | 692 | 670 | 59 |  |  |
| PEN3 | Bol021414v2 F | CGAGCTCTCTGTGCCTTTCG | 1347 | 1366 | 60 | 59 | 95 |
|  | Bol021414v2 R | CAGCTCTTGAGAAGCTCCATCTT | 1442 | 1420 | 58 |  |  |
| ESP1 | Bol006378 F | CTACACGACTGCTACCGTCTATGG | 921 | 944 | 59 | 58.5 | 70 |
|  | Bol006378 R | GGTTGTTGGTGGGACGTTTT | 991 | 972 | 58 |  |  |
| ESP2 | Bol039072v2 F | TGTTTGGACATGCGGTTGTG | 740 | 759 | 60 | 59 | 88 |
|  | Bol039072v2 R | CGTCCCTGGTCCCAAATG | 828 | 811 | 58 |  |  |
| ESM1 | Bol005067v2 F | TCCGATGTTGAACCAGTTTGC | 732 | 752 | 60 | 59.5 | 74 |
|  | Bol005067v2 R | CGAAGGATGGCGTTGTAGAAA | 806 | 786 | 59 |  |  |
| NSP1 | Bol036931v2 F | GTGTTGTTCGGAGGTGAGATAGC | 535 | 557 | 59 | 58.5 | 76 |
|  | Bol036931v2 R | GCAAAAGTCCCTTCGATCAATT | 611 | 590 | 58 |  |  |
| MVP1 | Bol035149 F | CGGGTCCGGAGGTAGGAA | 564 | 581 | 59 | 58.5 | 64 |
|  | Bol035149 R | TCACGGCCGGTAAGCAA | 628 | 612 | 58 |  |  |
| MBP2 | Bol035044v2 F | GGGAGAAACTTGAGGAAGGTCAT | 1682 | 1704 | 59 | 58.5 | 91 |
|  | Bol035044v2 R | ACCATACATCAACAGCCCCTTT | 1773 | 1752 | 58 |  |  |
| BAT5 | Bol030524v2 F | TGGTTTCTCACAACACTCGAAGA | 83 | 105 | 58 | 58.5 | 94 |
|  | Bol030524v2 R | GCGTAATGGAAAATCTACAGATGATG | 177 | 152 | 59 |  |  |

^a^The gene model used for the creation of each primer set is indicated in the primer name.

Primer sets created from cDNA sequences not included in Liu et al. [27] annotations that were chosen based on BLAST bit-score for similarity with A. thaliana annotated sequences are indicated in bold.
