# Supplementary figures and images for: Chemopreventive glucosinolate accumulation in various broccoli and collard tissues: Microfluidic-based targeted transcriptomics for by-product valorization
Source: PLoS One. 2017 Sep 25;12(9):e0185112. doi: 10.1371/journal.pone.0185112 (PMC5612653; doi:10.1371/journal.pone.0185112)

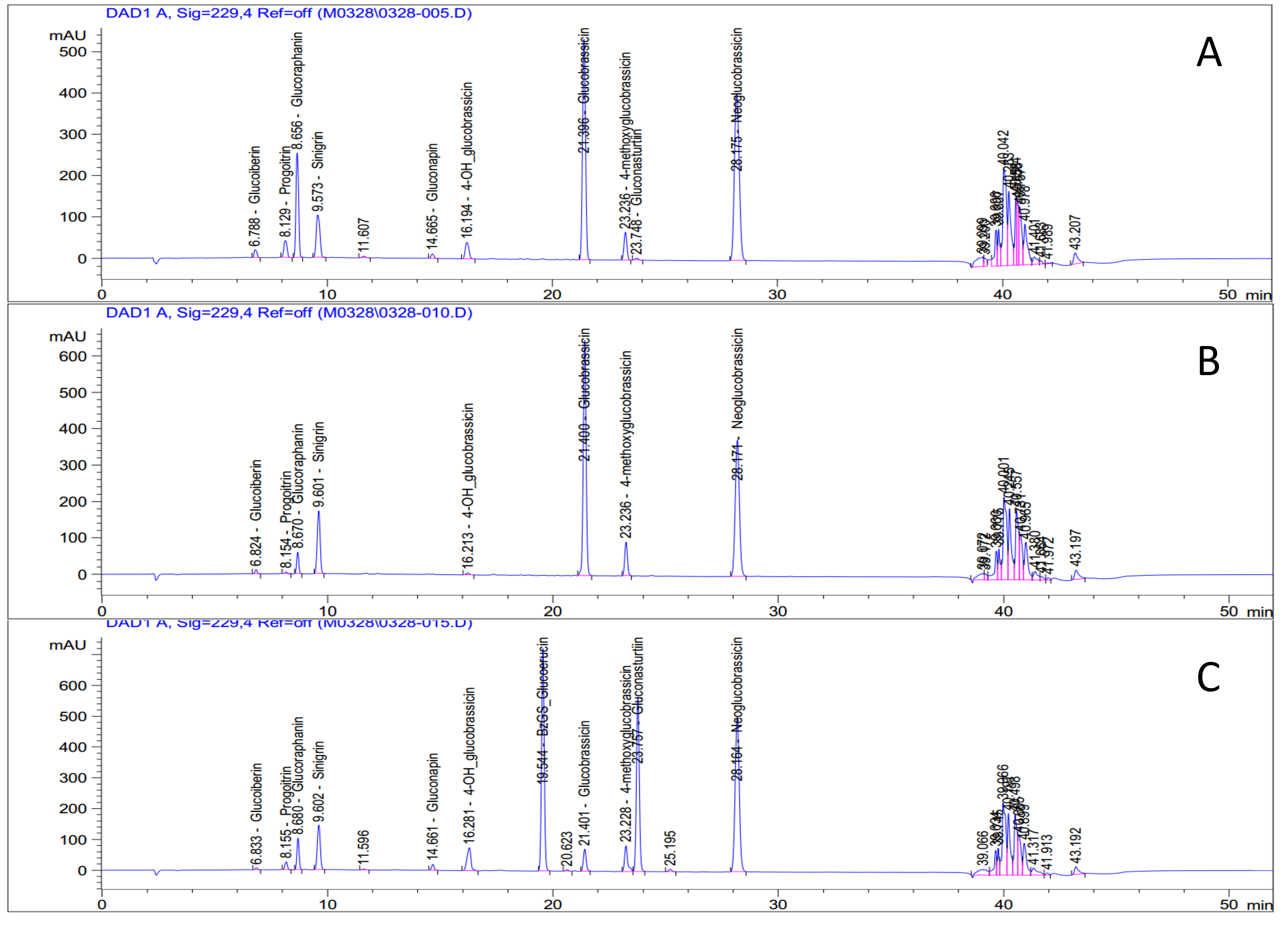

Supplement: S1 Fig — (TIF) [file pone.0185112.s008.tif]

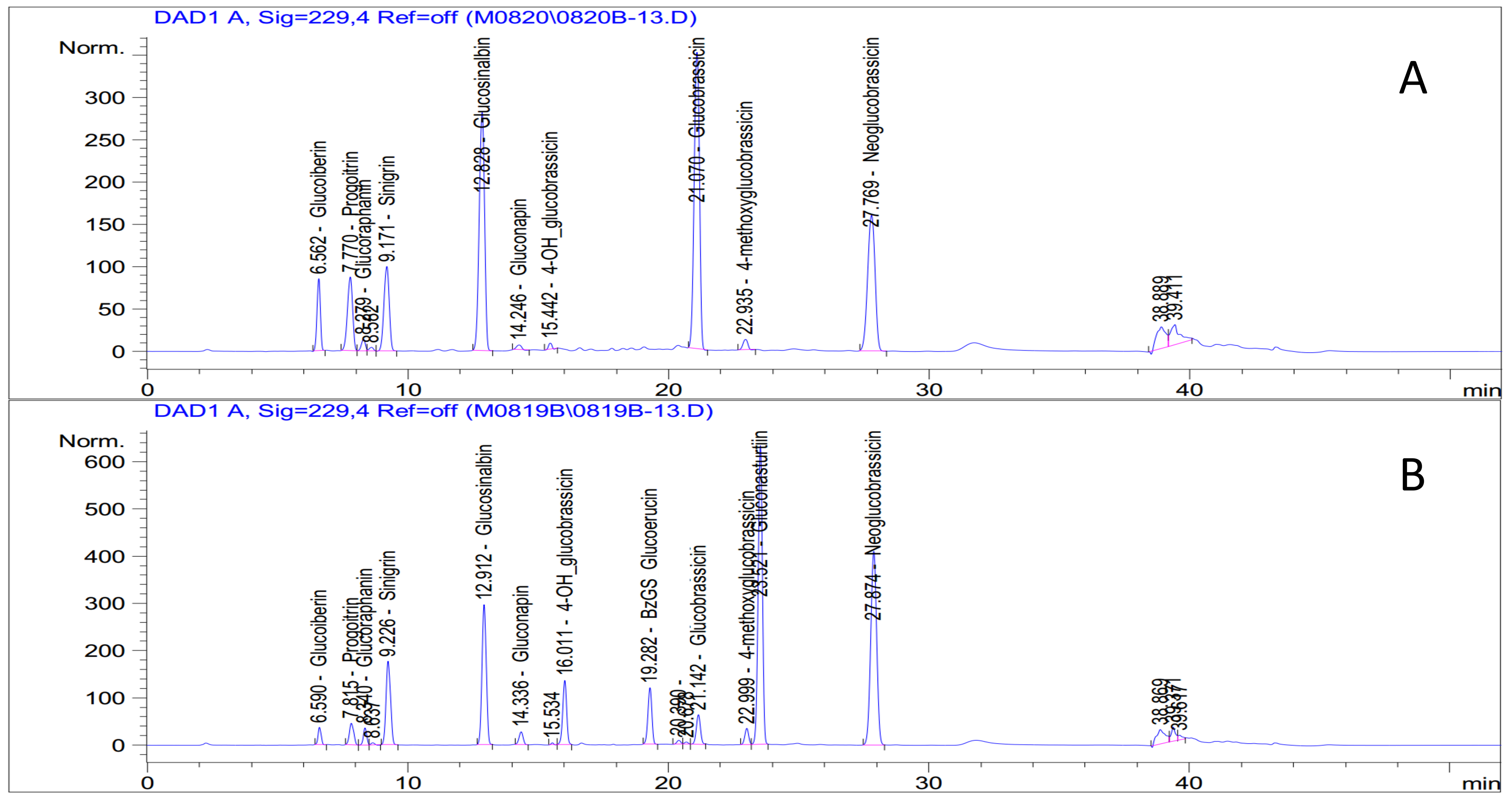

Supplement: S2 Fig — (TIF) [file pone.0185112.s009.tif]

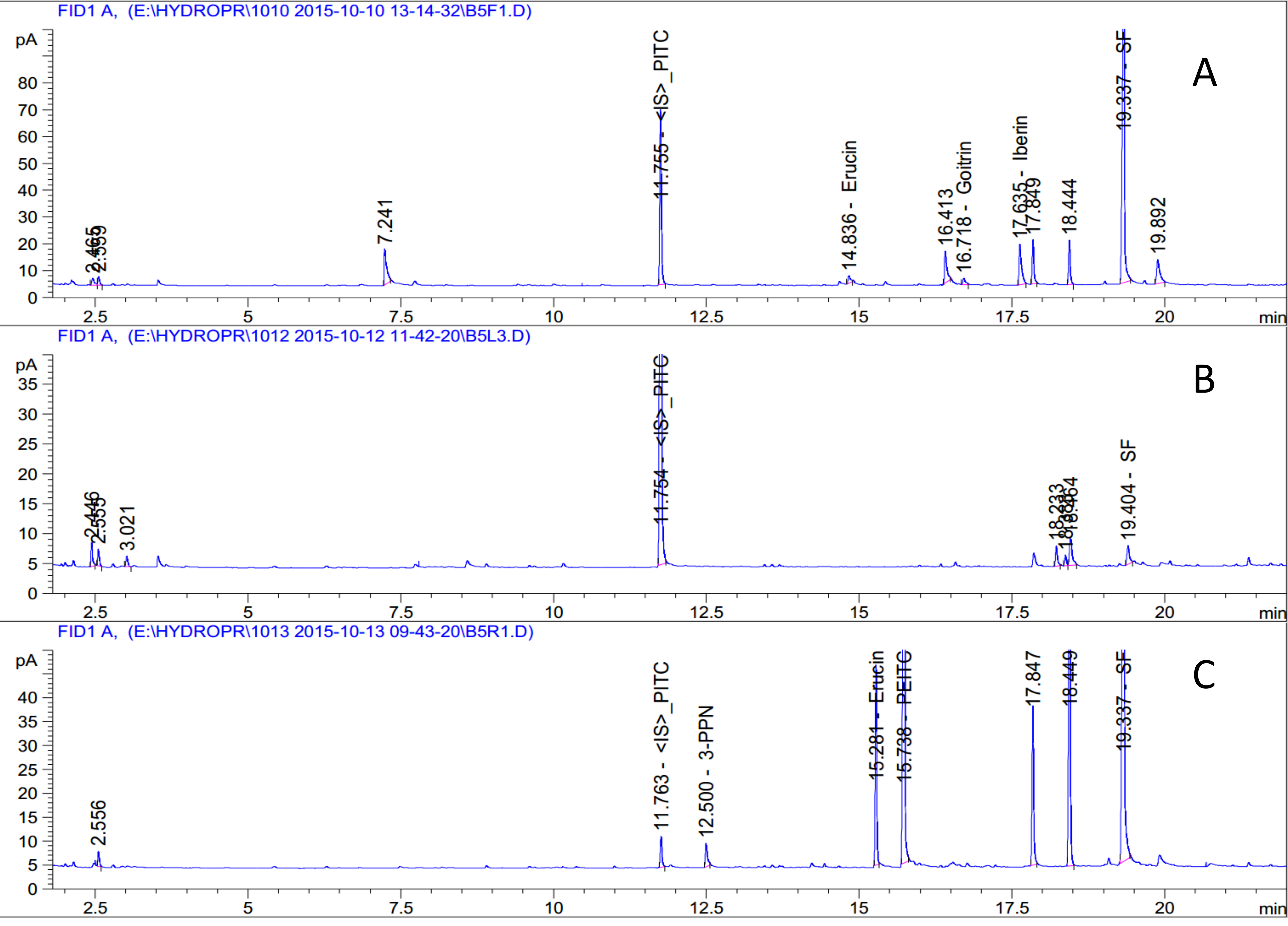

Supplement: S3 Fig — (TIF) [file pone.0185112.s010.tif]

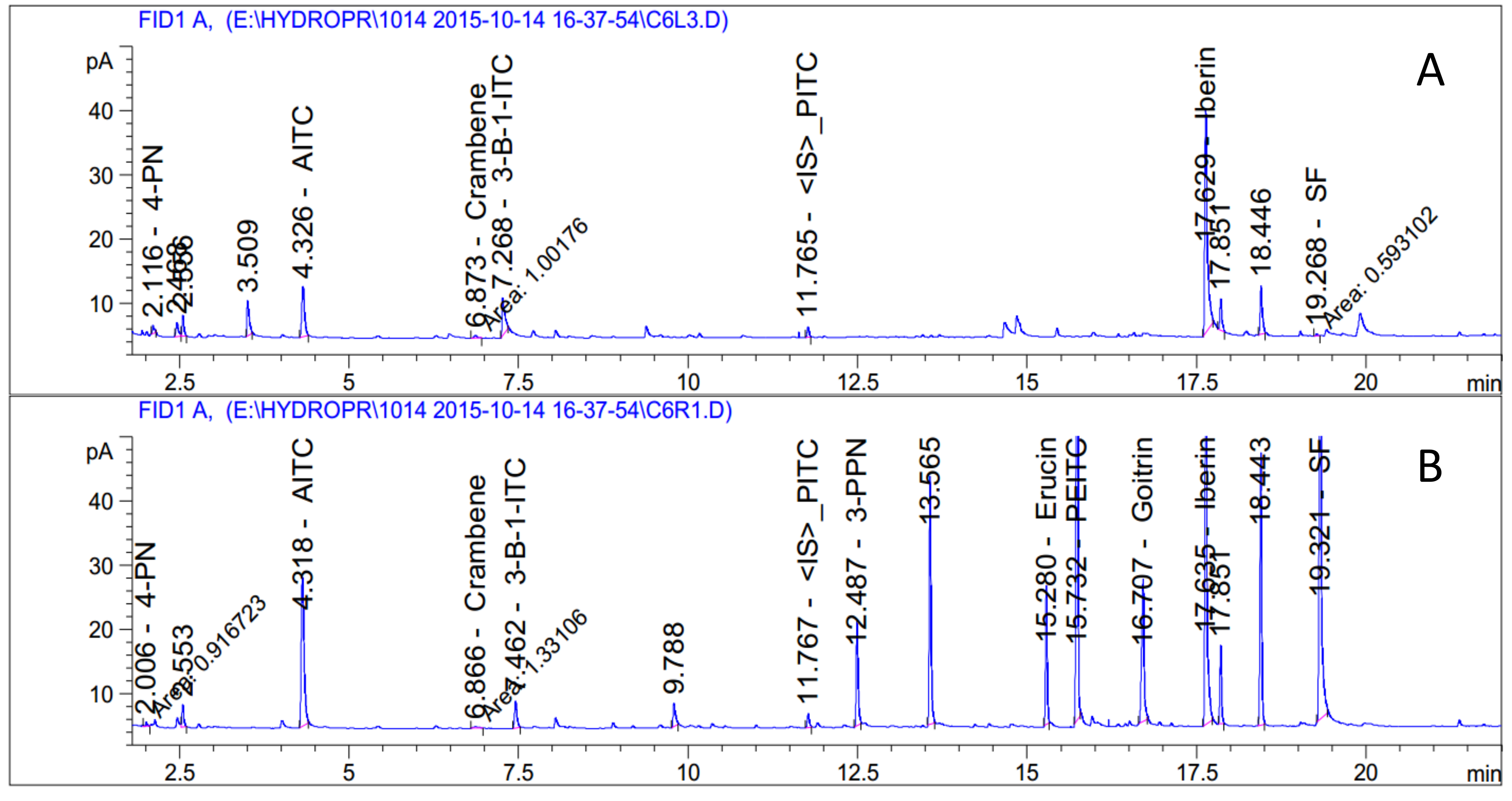

Supplement: S4 Fig — (TIF) [file pone.0185112.s011.tif]
